# Editorial: Unveiling the tumor microenvironment by machine learning to develop new immunotherapeutic strategies

**DOI:** 10.3389/fimmu.2023.1239648

**Published:** 2023-08-15

**Authors:** Ping Zheng, Jun Liu

**Affiliations:** ^1^Department of Neurosurgery, Pudong New Area People’s Hospital, Shanghai, China; ^2^Yuebei People’s Hospital, Shaoguan, China

**Keywords:** tumor microenvironment, machine learning, tumor, disease, immune infiltrations

A total of 35 paper are included in this series. We selected eight as representative ones:

Yang et al. reported pancreatic ductal adenocarcinoma (PDAC) with a high 7-methylguanosine (m7G) score were characterized by increased immune cell infiltration, increased genomic instability, higher response rate to combined immune checkpoint inhibitors (ICIs), and overall poor survival. Their findings indicate that the m7G score is associated with tumor invasiveness, immune cell infiltration, ICI treatment response, and overall patients’ survival. They also identified FN1 and ITGB1 as core genes in the m7Gscore model, which affect immune cell infiltration and genomic instability not only in pancreatic cancer but also in pan-cancer. FN1 and ITGB1 can inhibit immune T cell activation by upregulation of macrophages and neutrophils, thereby leading to immune escape of pancreatic cancer cells and reducing the response rate of ICI treatment.

Wang H. et al. reported that most of the 23 m6A regulators were significantly differentially expressed in the Esophageal cancer (ESCA) tissues. LASSO regression analysis was used to perform a prognostic risk model that included seven m6A-related regulators (FMR1, RBMX, IGFBP1, IGFBP2, ALKBH5, RBM15B, and METTL14). Moreover, they found that this risk model was significantly correlated with biological functions, including base metabolism, DNA repair, and mismatch repair. A nomogram was constructed to predict the prognosis of patients with ESCA. The results of bioinformatics analysis were further validated in human ESCA and normal tissues by qRT-PCR.

Cheng et al. applied an unsupervised cluster analysis based on Cyclin-dependent kinase inhibitor 2A (CDKN2A)-correlated genes unveiled three subtypes, namely cold-immune subtype, IFN-γ activated subtype and FTL-dominant subtype. Subsequent analyses depicting hallmarks of tumor microenvironment (TME) among three subtypes suggested strong association between triple-negative breast cancer (TNBC) and CDKN2A. Given the fact that the most clinically heterogeneous TNBC always displayed the most severe outcomes and lacked relevant drug targets, they further explored the potential of immunotherapy for TNBC by interfering CDKN2A and constructed the CDKN2A-derived prognostic model for TNBC patients by Lasso-Cox. The 21-gene–based prognostic model showed high accuracy and was verified in external independent validation cohort. Moreover, they proposed three drugs for TNBC patients based on our model *via* targeting epidermal growth factor receptor.

Wang W. et al. identified two subclusters based on cuproptosis-related signature (CRGs) in glioma. Patients in cluster2 had better clinical outcomes. The cuproptosis-signature was constructed based on CuproptosisScore. Patients with higher CuproptosisScore had higher WHO grades and worse prognosis, while patients with lower grades were more likely to develop IDH mutations or MGMT methylation. Univariate and Multivariate Cox regression analysis demonstrated CuproptosisScore was an independent prognostic factor. The accuracy of the signature in prognostic prediction was further confirmed in 11 external validation datasets. In groups with high-CuproptosisScore, PIK3CA, MUC16, NF1, TTN, TP53, PTEN, and EGFR showed high mutation frequency. IDH1, TP53, ATRX, CIC, and FUBP1 demonstrated high mutation frequency in low-CuproptosisScore group. The level of immune infiltration increased as CuproptosisScore increased. SubMap analysis revealed patients with high-CuproptosisScore may respond to anti-PD-1 therapy. The IC50 values of Bexarotene, Bicalutamide, Bortezomib, and Cytarabine were lower in the high-CuproptosisScore group than those in the low-CuproptosisScore group.

Xia et al. showed the high- Necroptosis-Related Gene Prognostic Score (NRGPS) group had significantly lower the overall survival (OS) than the low-NRGPS group in gastric cancer. Cox regression analyses showed that NRGPS was an independent prognostic variable. Tumor-mutation-burden (TMB), tumor microenvironment (TME), microsatellite instability (MSI), and Tumor Immune Dysfunction and Exclusion (TIDE) scoring were used as predictors of the immunotherapy response. The high-NRGPS group was characterized by a cancer-friendly immune microenvironment, a high TIDE score, and a low TMB, a low MSI all of which consistently demonstrated that the problems observed in the high-NRGPS group are associated with immune escape in gastric cancer GC.

Wang X. et al. detected Differentially expressed genes (DEGs) by the Wilcoxon test based on the TCGA-LGG dataset and the weighted gene co-expression network analysis (WGCNA) was implemented to identify the significant module associated with the expression level of FNDC3B. Furthermore, they investigated the correlation between FNDC3B with cancer immune infiltrates using TISIDB, ESTIMATE, and CIBERSORTx. Higher FNDC3B expression displayed a remarkably worse overall survival and the expression level of FNDC3B was an independent prognostic indicator for patients with glioma. Based on TCGA LGG dataset, a co-expression network was established and the hub genes were identified. FNDC3B expression was positively correlated to the tumor-infiltrating lymphocytes and immune infiltration score, and high FNDC3B expression was accompanied by the increased expression of B7-H3, PD-L1, TIM-3, PD-1, and CTLA-4. Moreover, expression of FNDC3B was significantly associated with infiltrating levels of several types of immune cells and most of their gene markers in glioma.

Xiao et al. applied the large-scale machine learning to find that SOX family can be divided into two distinct clusters in gliomas, with significant immune characteristics and genomic profiles. Among them, SOX10 was identified as an excellent immune regulator of macrophage in gliomas. High expression of SOX10 is related to shorter OS in LGG, HGG, and pan-cancer groups, but benefited from the immunotherapy. Single-cell sequencing proved SOX10 is high in neurons, M1 macrophages, and neural stem cells. Macrophages are found to be elevated in the SOX10 high expression group. SOX10 has a positive correlation with macrophage cytokine production and negative regulation of macrophages’ chemotaxis and migration.

Lu et al. created a cuproptosis-related lncRNA prognostic model based on the cuproptosis-related lncRNA score (CLS) by performing lasso regression. They identified ten cuproptosis-related genes and 13 correlated prognostic lncRNAs were collected for model construction. CLS was positively or negatively correlated with cancer-related pathways. In addition, cell cycle and immune related pathways were enriched. By performing tumor microenvironment (TME) analysis, they determined that T-cells were activated. High CLS had more tumor characteristics and may lead to higher invasiveness and treatment resistance. Three genes (TP53, CSMD1 and RB1) were found in high CLS samples with more mutational frequency. More amplification and deletion were detected in high CLS samples. In clinical application, a CLS-based nomogram was constructed. 5-Fluorouracil, gemcitabine and doxorubicin had better sensitivity in patients with high CLS. However, patients with low CLS had better immunotherapeutic sensitivity.

In summary, one study involved the single cell RNA-seq and the other study applied the digital spatial profiling. Other most studies were using the TCGA bulk-RNA seq data. The schematic flow can be summarized as follows (**Figure 1**): The authors first clustered the samples into two or three clusters based on the geneset or single gene medium expression. Then they established the diagnostic or prognosis models with machine learning methods and verified them in some cases with external datasets. At last, they compared the proliferation and invasiveness status, immune response with CIBERSORT, survival status and TMB, grades between or among these clusters. The future point would be adopting more advanced machine learning models, deep neural networks, transfer learning to deal with a big population data and compare the predictive ability of these machine learning methods.

**Figure 1:**
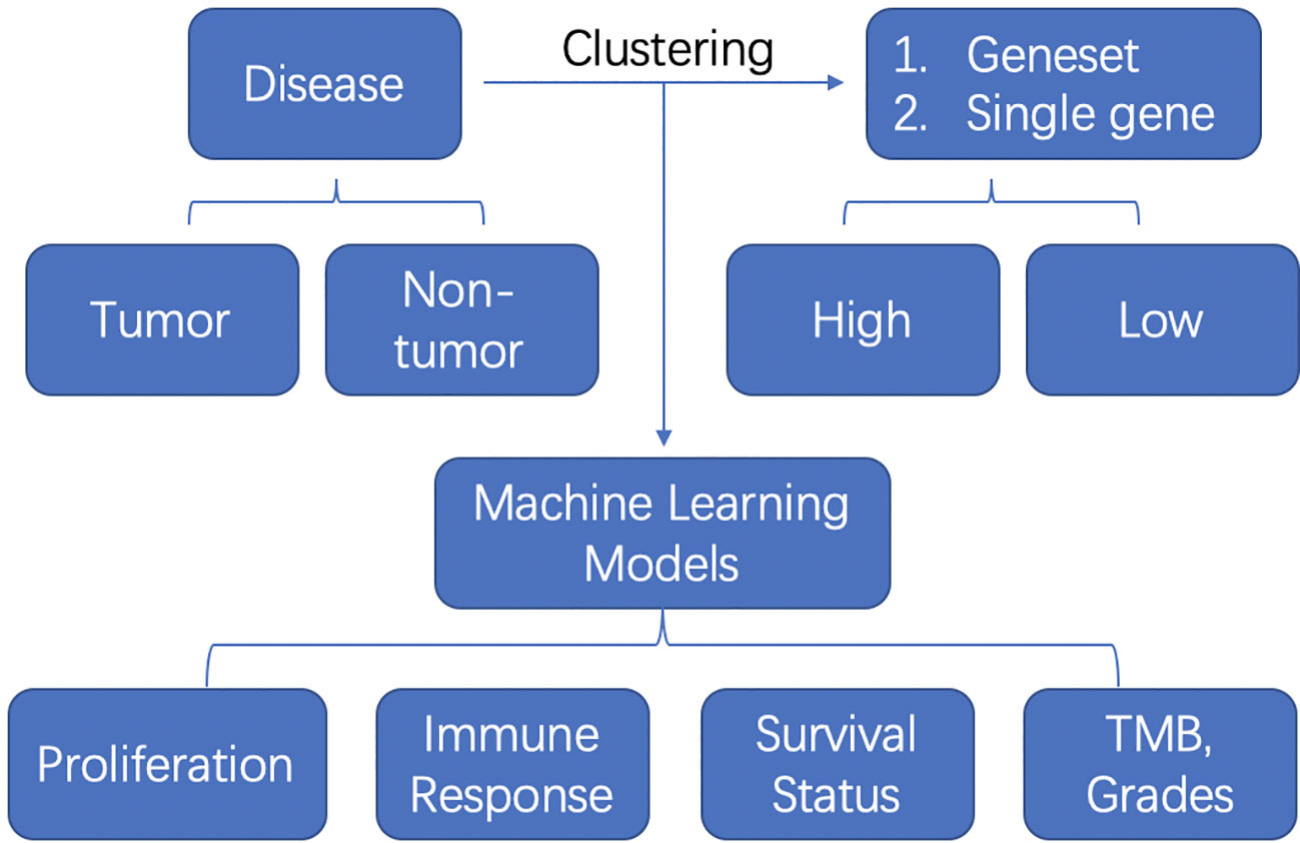
The schematic flow of machine learning model application in tumor or non-tumor diseases.

## Author contributions

All authors listed have made a substantial, direct, and intellectual contribution to the work and approved it for publication.

